# Differential developmental rates and demographics in Red Kangaroo (*Osphranter rufus*) populations separated by the dingo barrier fence

**DOI:** 10.1093/jmammal/gyad053

**Published:** 2023-05-31

**Authors:** D Rex Mitchell, Stuart C Cairns, Gerhard Körtner, Corey J A Bradshaw, Frédérik Saltré, Vera Weisbecker

**Affiliations:** College of Science and Engineering, Flinders University, GPO Box 2100, Adelaide, South Australia 5001, Australia; Australian Research Council Centre of Excellence for Australian Biodiversity and Heritage, Wollongong, New South Wales 2522, Australia; Centre for Behavioural and Physiological Ecology, University of New England, Armidale, New South Wales 2350, Australia; Centre for Behavioural and Physiological Ecology, University of New England, Armidale, New South Wales 2350, Australia; Australian Research Council Centre of Excellence for Australian Biodiversity and Heritage, Wollongong, New South Wales 2522, Australia; Global Ecology | Partuyarta Ngadluku Wardli Kuu, College of Science and Engineering, Flinders University, GPO Box 2100, Adelaide, South Australia 5001, Australia; College of Science and Engineering, Flinders University, GPO Box 2100, Adelaide, South Australia 5001, Australia; Australian Research Council Centre of Excellence for Australian Biodiversity and Heritage, Wollongong, New South Wales 2522, Australia; Global Ecology | Partuyarta Ngadluku Wardli Kuu, College of Science and Engineering, Flinders University, GPO Box 2100, Adelaide, South Australia 5001, Australia; College of Science and Engineering, Flinders University, GPO Box 2100, Adelaide, South Australia 5001, Australia; Australian Research Council Centre of Excellence for Australian Biodiversity and Heritage, Wollongong, New South Wales 2522, Australia

**Keywords:** geometric morphometrics, growth rates, interpopulation, intraspecific, population density, sexual dimorphism

## Abstract

Decommissioning the dingo barrier fence has been suggested to reduce destructive dingo control and encourage a free transfer of biota between environments in Australia. Yet the potential impacts that over a century of predator exclusion might have had on the population dynamics and developmental biology of prey populations has not been assessed. We here combine demographic data and both linear and geometric morphometrics to assess differences in populations among 166 red kangaroos (*Osphranter rufus*)—a primary prey species of the dingo—from two isolated populations on either side of the fence. We also quantified the differences in aboveground vegetation biomass for the last 10 years on either side of the fence. We found that the age structure and growth patterns, but not cranial shape, differed between the two kangaroo populations. In the population living with a higher density of dingoes, there were relatively fewer females and juveniles. These individuals were larger for a given age, despite what seems to be lower vegetation biomass. However, how much of this biomass represented kangaroo forage is uncertain and requires further on-site assessments. We also identified unexpected differences in the ontogenetic trajectories in relative pes length between the sexes for the whole sample, possibly associated with male competition or differential weight-bearing mechanics. We discuss potential mechanisms behind our findings and suggest that the impacts of contrasting predation pressures across the fence, for red kangaroos and other species, merit further investigation.

## Teaser

We show that one population of red kangaroos outside the dingo barrier fence has fewer females and joeys, and also grows up faster than a neighboring population that is protected by the fence. We argue that the impacts of contrasting predation pressures across the fence, for kangaroos and other prey, need further investigation.

**Fig. 1.**
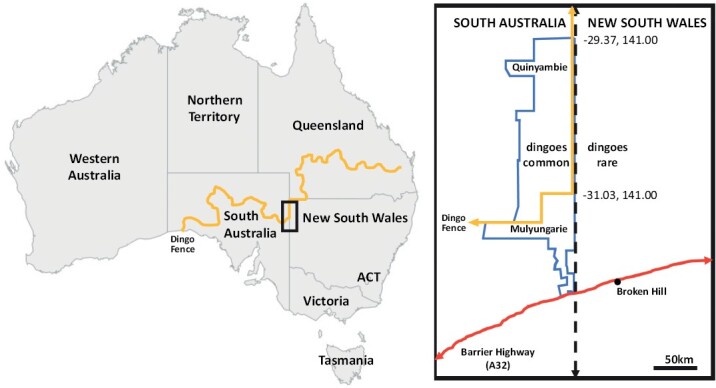
The dingo barrier fence extends from southeast Queensland to the Great Australian Bight in South Australia. The study location (black rectangle): Quinyambie and Mulyungarie stations are adjacent properties located on the western side of the South Australia–New South Wales border (black dotted line) and separated by the fence.

The dingo barrier fence in Australia is the longest environmental barrier in the world (Woodford 2003). At 5,614 km long, it exists as an exclusionary measure to control predation on sheep by dingoes (*Canis dingo* or *Canis familiaris*; cf. Jackson et al. 2019; Smith et al. 2019). Construction of parts of the ~1.7-m high fence began in the early 19th century and the barrier was formed in its entirety in the 1950s (Bauer 1964; Downward and Bromell 1990; Philip 2021). The structure now extends from southeastern Queensland west to southwestern South Australia (Fig. 1), thereby acting as an unintentional experiment in how predator exclusion affects ecosystems across much of the continent.

Extensive fencing can fragment habitats, alter species dispersal patterns, and disrupt ecosystem processes (Bradby et al. 2014; Jakes et al. 2018). Over the last decade, many studies have revealed that the exclusion of an apex predator can substantially alter ecosystem balance and function on either side of the dingo barrier fence. For example, dingo exclusion by the fence has been linked to shifts in the structure of mammalian communities (Letnic and Koch 2010; Rees et al. 2019; Mills et al. 2021), ground-dwelling marsupial extinctions resulting from increases in mesopredator abundance (Johnson et al. 2007), and changes to floral assemblages (Gordon et al. 2017; Rees et al. 2017; Fisher et al. 2021). These effects have elicited far-reaching trophic cascades, and also altered soil nutrient profiles (Morris and Letnic 2017) and landscape geomorphology (Lyons et al. 2018).

Maintenance of the dingo barrier fence is expensive, estimated a decade ago at AU$10 million per year (Bradshaw and Ritchie 2012). Further, associated control measures—such as trapping and poisoning of dingoes along its margins—can be detrimental to nontarget, native species (Philip 2021). Decommissioning the fence has therefore been suggested to reduce dependence on trapping and poisoning, and to promote biodiversity and healthier ecosystem function (Philip 2021). However, the impact of ~100 years of predator exclusion on the population dynamics and developmental biology of prey species has received little attention. Anticipating the response of prey populations to release from dingo predation is important for predicting the effects of opening or moving the fence. In addition, the dingo barrier fence offers an opportunity to test hypotheses regarding how populations of mammalian herbivores respond to shifts in predation, in relation to herbivore conservation, and inference of past ecosystems.

The largest extant native herbivore in Australia, the red kangaroo (*Osphranter rufus*), is directly affected by the dingo barrier fence because it is a primary prey species of the dingo (Shepherd 1981; Marsack and Campbell 1990). Commonly found in the semiarid and arid zones across Australia (Richardson 2012; Freedman et al. 2020), geographically isolated populations exist on either side of the dingo barrier fence, sometimes within few kilometers of each other (Caughley et al. 1980; Pople et al. 2000). Red kangaroos tend to be more abundant where dingo control is highest (Dawson et al. 2022), and differences in red kangaroo population densities on either side of the fence are well-documented (Caughley et al. 1980; Pople et al. 2000; Letnic and Koch 2010; Morris and Letnic 2017). However, it is unclear whether these differences are due directly to predation by dingoes or attributable to vegetation change and water availability across the fence (Caughley et al. 1980; Newsome et al. 2001). The combination of contrasting predation and divergent habitats on either side of the dingo barrier fence therefore makes the red kangaroo a good candidate to assess demographic and morphological responses during short-term ecological changes. Understanding how demographics and morphology can shift in relation to predation pressure also has the potential to provide insights on the processes that led to the extinction of some megafauna (Johnson et al. 2016; Saltré et al. 2019; Bradshaw et al. 2021).

While demographics can be assessed in *O. rufus* via age distributions (estimated from molar progression indices; Kirkpatrick 1964) and sex ratios, a generalized proxy of vegetation differences can be ascertained by: (1) changes in satellite-derived time series of enhanced vegetation index data (index of greenness, so that higher enhanced vegetation indicates greater food biomass available to kangaroos; Matsushita et al. 2007), and (2) interpopulation differences in the shape of the cranium, which we can investigate with three-dimensional landmark coordinate data using geometric morphometrics (Weisbecker et al. 2019; Mitchell et al. 2020).

Here we evaluate the impact of the dingo barrier fence on red kangaroo demography (i.e., sex ratio and age structure) in terms of developmental (ontogenetic) growth rate, and cranial morphology. Specifically, we present demographic data (sex, age, body weight, and pes length) and information on the shape of the upper skull (crania) of 166 red kangaroos randomly sampled from either side of the dingo barrier fence in 2018, in addition to comparing the variation in enhanced vegetation index over the last 10 years on either side of the fence, to test the three following hypotheses: (1) the population living with greater dingo densities has lower population density, and fewer females, juveniles, and old individuals than the population living where dingoes are rare, given the evidence of dingoes selecting smaller prey (Shepherd 1981) and a presumed increase in prey susceptibility with senescence (Hastings 1984); (2) because either dingo predation or environmental differences are expected to reduce population size outside the dingo barrier fence (Caughley et al. 1980; Newsome et al. 2001), the population living with greater dingo densities has higher average ontogenetic growth rates at a lower density (Plaisir et al. 2022); and (3) a more diverse and higher-biomass vegetation community with more dingo presence (given lower browsing and grazing pressure by kangaroos) would alter cranial morphology in response to biomechanical requirements of feeding (Weisbecker et al. 2019; Mitchell et al. 2020). We also used this data set to analyze intraspecific variation and developmental sexual dimorphism in *O. rufus*, which is useful information for future age and sex determination from dry specimens. While only a single sampling point in time, our study is the first to produce a comprehensive assessment of cross-dingo fence differences in demographics, growth, and morphology of a dingo prey species.

## Materials and Methods

The study site includes two adjacent properties separated by the dingo barrier fence (Fig. 1). The Mulyungarie property (31°25ʹ59″S, 140°47ʹ49″E) is located south of the fence and is delimited to the east by the border between New South Wales and South Australia, and to the south by the Barrier Highway. The Quinyambie property (30°21ʹ43″S, 140°41ʹ46″E) is to the north of Mulyungarie, also bordered to the east by the State boundary. While dingoes are present on both properties, the densities differ greatly so that dingoes are considered common in Quinyambie, but rare in Mulyungarie (Mills et al. 2021). For brevity, we refer to Quinyambe as ‘dingoes-common’ and Mulyungarie as ‘dingoes-rare’ in the analysis and interpretations. The two properties are directly adjacent to one another. While the fence in this region was in poor condition in the 1950s, it was restored as an effective barrier by 1975 (Leane 1996). Assuming an average of 30 months for female red kangaroos to reach sexual maturity (Frith and Sharman 1964), we estimate a minimum of ~17 generations since frequent dingo exposure to the time of data collection, although it could be more.

The sample was collected for a longer-term research project led by SCC and GK (under Animal Ethics Permit AEC 17-102 from the University of New England), with a South Australian Department of Environment and Water Permit to Undertake Scientific Research (K12941-27). The red kangaroos from Mulyungarie and Quinyambie Stations were shot by a professional kangaroo shooter during an annual shooting operation as part of a longer-term population assessment. All animals encountered during nocturnal searches were collected, such that the sample should represent a random selection of relative densities, sex ratios, and age structures from this time. All were either neck or chest shot, then exsanguinated in accordance with the code of practice for shooting kangaroos for noncommercial purposes (Commonwealth of Australia 2008). The exsanguinated carcasses were weighed using electronic scales, sexed, and the length of the right hind pes measured to the nearest cm including the claw (Jarman 1989). Heads were removed from the carcasses and rendered in boiling water for 6–8 h to facilitate defleshing to obtain a clean skull for age determination via molar progression (Kirkpatrick 1964; Hadley et al. 2009). We determined the molar index (*M*) by measuring the number of molars that had progressed past the posterior rims of the zygomatic arches (Kirkpatrick 1964; Death and Coulson 2016). We then estimated the age in days (*A*) using the formula for aging red kangaroos in Kirkpatrick (1970):


$$\begin{array}{l} \log_{e}A=2.2278+0.359M \end{array}$$ (1)

To test for differences in sex ratio between the two samples, we did a chi-squared test using the *chisq.test* function in R (McHugh 2013).

We landmarked the crania using a Microscribe (Immersion Corporation, San Jose, California). The landmark scheme follows Mitchell et al. (2018). We analyzed landmarks and all other data using the R (R Core Team 2022) package Geomorph V3.3.1 (Adams and Otárola-Castillo 2013; Adams et al. 2021). We did a Procrustes superimposition on the raw shape coordinates with the *bilat.symmetry* function in Geomorph. This removed any variation attributable to scale, position, orientation, and morphological asymmetry (Rohlf and Slice 1990; Klingenberg et al. 2002). Given the sexual dimorphism of this species (Jarman 1989), we tested females and males from the two properties separately for all interpopulation tests. We tested whether males and females on each side of the fence differed in widely used metrics of size including cranial centroid size (derived from the cranial landmarks), body weight, and pes length. To examine the interrelationships among these size metrics, we regressed pes length and cranial size. To test for differences in cranial shape between populations, we constructed a multivariate Procrustes linear model of shape using the *procD.lm* function in Geomorph at 1,000 permutations with age and population as predictors.

To test for sexual dimorphism in ontogenetic growth rates in the whole population, we assessed how age predicted cranial size, body weight, pes length using the ‘nlme’ R package based on code adapted from Carlisle et al. (2017). For age-based analyses, we applied a von Bertalanffy model typical of animal growth (von Bertalanffy 1938, 1957) of the form:


$$\begin{array}{l} L_{t}=L_{\infty }(1-e^{-k(t-t_{0})}) \end{array}$$ (2)

where *L*_*t*_ = length at time (age) *t*, *L*_∞_ = asymptotic maximum length, *k* = the growth coefficient, and *t*_0_ is the theoretical age at length = 0 (Beverton and Holt 1959). We tested whether *L*_∞_, *k*, or *t*_0_ differed between the sexes, but focused mainly on whether each sex reached *L*_∞_ at different sizes, indicating sexual dimorphism in final adult size.

We also tested for differences in ontogenetic allometry (size-related developmental effects; Klingenberg 1996) based on cranial shape (Procrustes residuals) across the species using the *procD.lm* function with 1,000 permutations and the natural logarithm of centroid size as a predictor. The code and raw 3D data are available at https://github.com/DRexMitchell/Mitchell-et-al.-DIngo-Fence.

To test for difference in vegetation cover on each side of the fence, we used the enhanced vegetation index at each property location between 2011 and 2021. This remotely sensed index captures the greenness of the vegetation within an area and is a useful tool to assess the primary productivity of the ecosystem through its relationship with aboveground biomass production (Sims et al. 2006). The enhanced vegetation index is designed to enhance the vegetation signal with improved sensitivity in high biomass regions and improved vegetation monitoring through a decoupling of the canopy background signal and a reduction in atmosphere influences. This index tends to be more sensitive to plant canopy differences such as leaf area index, canopy structure, and plant phenology. We downloaded the enhanced vegetation index from the moderate-resolution imaging spectrometer (MODISv6; modis.gsfc.nasa.gov) from January 2011 to December 2021. The MODIS-enhanced vegetation index is available at a 250 × 250 m spatial resolution at 16-day intervals across the entire period to eliminate most of the cloud cover in daily images. We set any grid cell with an enhanced vegetation index < 0 to zero because it indicates the absence of vegetation within the grid cell. We corrected the index for the seasonal component due to phenology through time (De Keersmaecker et al. 2015) by calculating the scaled enhanced vegetation index anomaly (∆_EVI_) for each datum (Goetz et al. 2006) such that:


$$\begin{array}{l} \Delta_{EVI}(i,t)=\frac{\left(EVI(i,t)-mean_{u\in m}[EVI(i,u)]\right)}{sd_{u\in m}[EVI(i,u)]} \end{array}$$ (3)

where EVI(i,t) is the Enhanced Vegetation Index of grid cell *i* at date *t*, *m* is a month of the year and $mean_{u\in m}[EVI(i,u)]$ and $sd_{u\in m}[EVI(i,u)]$ are the mean and the standard deviation of the index for grid cell *i* over all dates *u*, across the entire period (2011–2021) falling within month *m*, respectively. The anomaly is unitless and on a scale where the interval in 1 *SD* of the enhanced vegetation index from the grid cell and the month in question. An anomaly of zero represents a baseline value of productivity. We then defined a temporal moving algorithm that we applied to ∆_EVI_ to reduce the effect of noise from clouds or aerosols (De Keersmaecker et al. 2015) by calculating the average index anomaly within the window *W*, such that:


$$\begin{array}{l} W(i,t)=\frac{\overset{5}{\underset{\tau =0}{\sum }}\Delta_{EVI}(i,t+8\tau )}{6} \end{array}$$ (4)

where *τ* indicates the number of MODIS data spanning six MODIS time intervals (96 days), with the magnitude of *W* having no effect on the average enhanced vegetation index anomaly (White et al. 2020).

To test whether the difference in the median of ∆_EVI_ for 2011–2021 on each side of the fence did not arise by chance, we compared the difference in the median of ∆_EVI_ between the two properties ($\Delta_{EVI_{\left(obs\right)}}$) with the distribution of thousands of median differences $\Delta_{EVI_{\left(rand\right)}}$ obtained by randomly reordering the data (Manly and Navarro Alberto 2020). If $\Delta_{EVI_{\left(obs\right)}}$ is the result of a random process, then all possible randomized orders of the data were equally likely to have occurred and with the same probability as the observed data. If the median difference in ∆_EVI_ between each side of the fence is not a random process, $\Delta_{EVI_{\left(obs\right)}}$ should appear as a typical value from the randomized distribution of thousands of $\Delta_{EVI_{\left(rand\right)}}$. The randomization procedure followed these steps: (1) we estimated $\Delta_{EVI_{\left(obs\right)}}$ based on our data; (2) we randomly reattributed (via permutation) the ∆_EVI_ of each time interval to a different time interval among the two sites and to estimate a new $\Delta_{EVI_{\left(rand\right)}}$; (3) we repeated step 2 10,000 times and thus obtained 10,000 $\Delta_{EVI_{\left(rand\right)}}$; and (4) we estimated a confidence interval for complete randomness by calculating the 2.5th and 97.5th percentiles of the $\Delta_{EVI_{\left(rand\right)}}$ distribution and calculate the probability that $\Delta_{EVI_{\left(obs\right)}}$ is a value in the distribution of $\Delta_{EVI_{\left(rand\right)}}$.

## Results

### Interpopulation analyses.

**Fig. 2.**
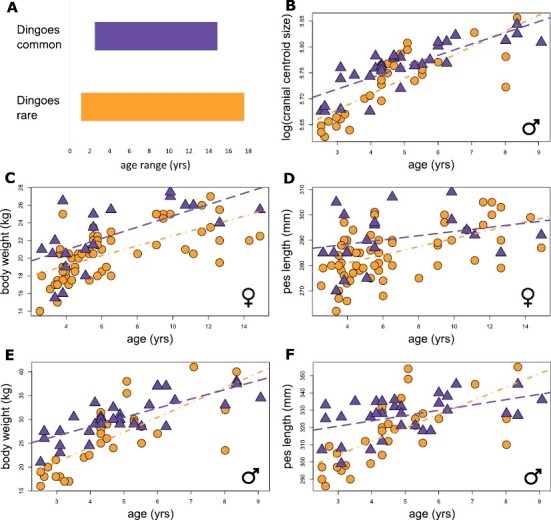
Comparisons of populations of red kangaroos where dingoes are rare (circles) and dingoes are common (triangles): (A) the dingoes-common population has a narrower range of ages. We excluded dingoes-rare individuals outside the age range of the dingoes-common population for subsequent analysis; (B) male cranial size per age; (C) female weight per age; (D) female pes length per age; (E) male weight per age; (F) male pes length per age.

Despite identical collection methods, there were fewer kangaroos collected on the dingoes-common property (*n* = 51) than on the dingoes-rare property (*n* = 115). There was a large female bias ($\chi_{1}^{2}$= 15.32, *P* < 0.001) in the dingoes-rare population (67.8%; 78 females) compared to the dingoes-common population (39.2%; 20 females). The age structure of the dingoes-rare population entirely enveloped that of the dingoes-common population (Fig. 2A). Given these different ranges of ages between the two populations, we excluded individuals from the dingoes-rare population that extended beyond the age range of the dingoes-common population for all subsequent analyses to focus on equivalent age ranges. This excluded 17 individuals below and five individuals above the age range of the dingoes-common population.

We found no evidence of differences in cranial shape between the populations for either sex (Table 1), but younger dingoes-common males had larger crania than younger dingoes-rare males (Fig. 2B). While there was substantial overlap for females between populations, dingoes-common females were heavier (Fig. 2C) and had a longer pes at a given age (Fig. 2D) than those from the dingoes-rare population. Similarly, dingoes-common younger males also tended to be heavier (Fig. 2E) and had a longer pes (Fig. 2F) than those from the dingoes-rare population. But in most instances, these trends decreased in magnitude with age and tended to dissipate between the populations in older males as indicated by the support for an interaction term between age and population for pes length (Fig. 2F).

**Table 1.** Interpopulation permutational linear model tests between dingoes-rare (Mulyungarie) and dingoes-common (Quinyambie) populations: “population” = the two populations; “age” is in years.

		Female			Male		
Model		*R* ^2^	*F*_1,79_	*P*	*R* ^2^	*F*_1,59_	*P*
Cranial shape	Age	0.104	9.39	0.001	0.117	7.94	0.001
	Population	0.018	1.59	0.068	0.020	1.08	0.145
Cranial size	Age	0.645	142.52	0.001	0.621	114.00	0.001
	Population	0.002	0.040	0.534	0.047	8.68	0.006
Body weight	Age	0.384	56.04	0.001	0.510	72.75	0.001
	Population	0.075	10.89	0.003	0.077	10.970	0.002
Pes length	Age	0.199	20.98	0.001	0.329	34.85	0.001
	Population	0.051	5.33	0.031	0.063	6.69	0.011
	Age:population				0.060	6.35	0.014

### Intraspecific analysis.

**Fig. 3.**
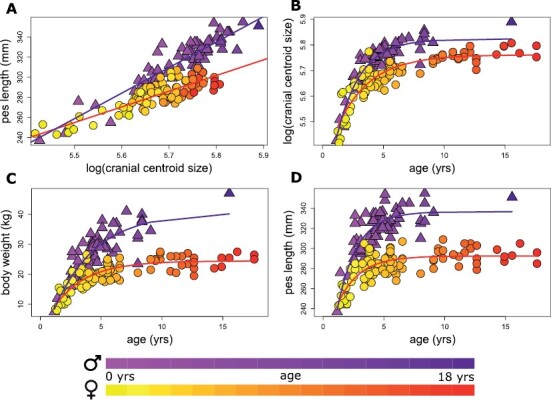
Intraspecific variation across the entire sample of *Osphranter rufus*. Males are represented by triangles, females by circles: (A) regression of pes length on cranial size indicates sexual dimorphism in relative pes length; (B) sexual dimorphism in cranial ontogenetic growth; (C) sexual dimorphism in the ontogeny of body weight; (D) sexual dimorphism in the ontogeny of pes lengths.

There was evidence for an interaction between cranial size and sex in pes length, indicating different allometric trajectories in pes length between the sexes (Fig. 3A). For a given cranial centroid size, male pes length increased faster than for females. Asymptotic maxima of the von Bertalanffy growth curves also differed between the sexes for cranial size, body weight, and pes length, with the estimated curves diverging for all three characters at approximately 2–2.5 years of age (Fig. 3B–D).

**Fig. 4.**
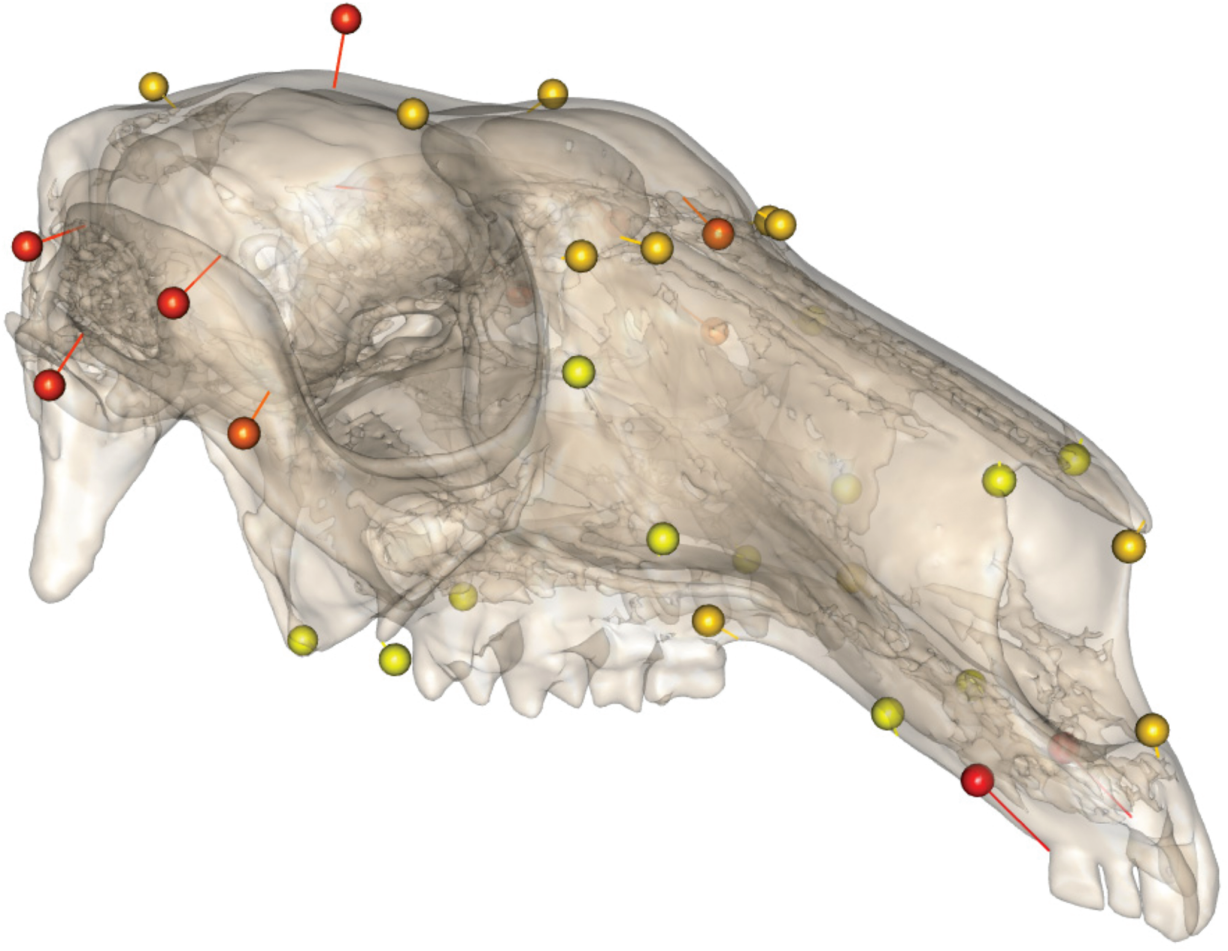
Changes in cranial shape throughout ontogeny in *Osphranter rufus*. Orbs represent cranial shape predicted for younger individuals. Mesh represents shape predicted for older individuals. Relative braincase size and incisor size become smaller during growth.

Cranial shape varied with centroid size (*R*^2^ = 0.21, *P* = 0.001; Table 2), with most of this ontogenetic allometry affecting relative braincase size and incisor size/orientation (Fig. 4). There was only a small interaction effect between sex and size (*R*^2^ = 0.01, *P* = 0.001; Table 2), with little visual differences discernible given the small effect size.

**Table 2.** Intraspecific tests: permutational regressions of linear models for cranial shape and pes length, followed by von Bertalanffy growth curves (nonlinear models) for cranial size, body weight, and pes length. *L*_∞_ = asymptotic size, *k* = growth coefficient, *t*_0_ = estimated age when size is zero.

Linear models	*R* ^2^	*P*
Cranial shape		
Log(centroid size)	0.210	0.001
Sex	0.020	0.001
Log(centroid size):sex	0.010	0.005
Pes length		
Log(centroid size)	0.668	0.001
Sex	0.201	0.001
Log(centroid size):sex	0.025	0.001
Nonlinear models	*t*_159_	*P*
Cranial size ~ age		
* L*_∞_	4.22	<0.001
* k*	2.01	0.047
* t*_0_	2.52	0.013
Body weight ~ age		
* L*_∞_	6.93	<0.001
* k*	−1.07	0.285
* t*_0_	0.90	0.371
Pes length ~ age		
* L*_∞_	11.14	<0.001
* k*	0.43	0.669
* t*_0_	1.43	0.154

### Environmental analysis.

**Fig. 5.**
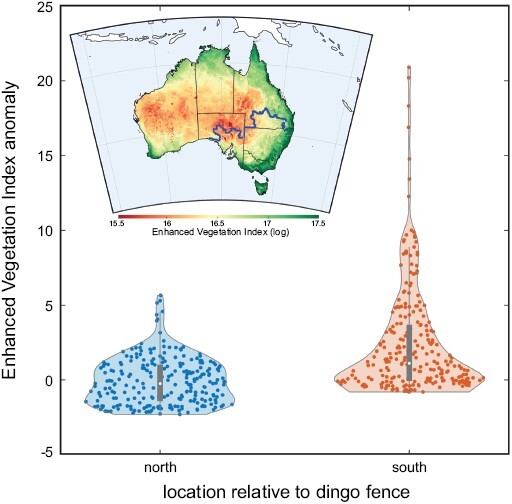
Spatial variation of vegetation greenness in Australia. Violin plots representing the difference in enhanced vegetation index anomalies from 2011 to 2021 between the dingoes-common property (north, left) and the dingoes-rare property (south, right). Each boxplot (dark gray) shows the median (white circle) and quartiles (box limits = 0.25 and 0.75, respectively) with whiskers reaching up to 1.5 times the interquartile range and the violin plot outlines illustrate kernel probability density (width of the shaded area represents the proportion of the data located there). Inset: median enhanced vegetation index (calculated over 2011–2021 time period) across Australia ranging from low greenness (characterizing barren/rocky ecosystem or grassland) to high greenness values (indicating open/closed forest).

Our results show that the enhanced vegetation index describes the spatial distribution of vegetation in Australia well (Fig. 5, inset), with higher enhanced vegetation index values (log of the index > 17) along the eastern and northern coast (characterizing both closed and open forests) compared to central Australia dominated by shrubs and grasslands (log of the index < 16). Our environmental analysis showed a scaled enhanced vegetation index anomaly south of the dingo fence (dingoes-rare population) of ~1.34 (unitless, but expressed as an *SD* of the index at a given location) higher than at the north of the fence (dingoes-common; Fig. 5). The productivity at the south of the fence presents a much higher variability (from −0.04 to 3.6, based on the 0.25 and 0.75 quartiles, respectively, with a maximum at 20.91) than in the north, which ranged from −1.40 to 0.99 (0.25 and 0.75 quantiles, respectively) with a maximum value at 5.6. Our randomization test indicates that the probability of the difference in median-scaled enhanced vegetation index anomalies between the north and the south of the fence could occur by chance was <0.001.

## Discussion

The dingo barrier fence provides an opportunity to examine the impact of contrasting selective pressures from similar localities on the intraspecific population biology of the largest living native herbivore in Australia, the red kangaroo. We showed that a population of red kangaroos from outside (north) of the fence, where dingoes are more common, grew faster, achieving larger sizes at younger ages, and contained fewer juveniles and females than a population from inside (south) of the fence where dingoes are rare. Our use of diverse body-size indicators also unexpectedly revealed that male red kangaroos grow a relatively longer pes than females with increasing size, providing a quantifiable basis for assessing males and females separately when using pes lengths in age and demographic assessments.

Several of our results independently support either increased ontogenetic growth rates in the dingoes-common population, slowed growth rates in the dingoes-rare population, or both. Females and younger males from the dingoes-common population were heavier with greater pes lengths at a given age. Younger males from the dingoes-common population also developed larger crania at a younger age. These differences were clearest in all cases for individuals younger than approximately 5 years. The clearer divergence among younger males is likely a result of the higher emphasis placed on growth in males compared to females (Promislow 1992). Dingo selection of younger and lighter prey (Shepherd 1981) might provide a plausible mechanistic explanation for increased sizes in younger individuals. Alternatively, these differences could arise due to low population densities (such as those in dingoes-common populations; see below) associated with increased bone growth in juveniles (Plaisir et al. 2022). However, the results of Plaisir et al. (2022) derived from a limited age range (21.2–29.0 months; mean = 26 months) for grey kangaroos (*Macropus giganteus*) were less pronounced than our observations from a broader age representation, suggesting that a direct impact through prey selection remains a plausible mechanism for the pattern we observed in addition to potential population density effects.

We cannot definitively conclude whether size differences between younger individuals across populations result from survival bias. It is possible that the faster-growing individuals were those that had evaded predation until collection for this study. However, we found that the size measurements of younger dingoes-common individuals were substantially higher than those of the dingoes-rare population, especially in young males, which instead suggests that ontogenetic growth rates have slowed on average following generations without regular exposure to dingo predation. We are also unable to distinguish whether these increased growth rates are a genetically selected trait, or a somatic (i.e., nonheritable and plastic) response to predation pressure. Higher growth rates have been observed as a somatic response in prey species after the introduction of predator cues (Bell et al. 2011). If the differential growth rates are associated with predation risk, a somatic response is likely reversible over short time frames, but a genetic response could have longer-lasting effects on population biology following the introduction of novel conditions (i.e., in this context, relocation or removal of the dingo fence). Intraspecific competition alongside a reduction in available forage from increased densities might also play a role in slowed development (Narvaez et al. 2020).

The dingoes-common population had fewer individuals, with a notable absence of ages below ~3 years, and a skewed sex distribution in favor of males. The density of dingoes-rare *O. rufus* south of the dingo fence was also an order of magnitude greater than the dingoes-common population and mirrors previous findings (Caughley et al. 1980; Pople et al. 2000; Letnic and Koch 2010; Morris and Letnic 2017). Caughley et al. (1980) found higher red kangaroo densities on the northern New South Wales side of the fence compared to the southern Queensland and northern South Australia side and concluded that differences in population density were attributable mainly to dingo predation. Despite our data supporting this conclusion, the causal mechanisms behind shifts in population densities remain elusive. For example, rainfall and forage biomass can affect pouch young (Newsome et al. 2001) and juvenile survival (Plaisir et al. 2022).

Comparing the vegetation productivity (scaled enhanced vegetation index anomalies; Fig. 5) over the last 10 years on either side of the barrier appears to support an influence of predation on growth rates, because the dingoes-rare zone south of the fence had higher vegetation productivity (i.e., greener/chlorophyll-based vegetation) than the dingoes-common zone north of the fence. However, this conclusion assumes that an increase in vegetation productivity indicates higher food availability for red kangaroos. If this is the case, we would have expected faster ontogenetic growth rates in the dingoes-rare property relative to the dingoes-common one. Our results show the opposite, which would rule out an environmental determinant as the principal mechanism modifying kangaroo population growth rates. Furthermore, while Plaisir et al. (2022) found increased ontogenetic growth rates at lower densities, their result was only clearly evident when there was high forage biomass. Instead, we found faster growth rates in the population with lower total forage biomass. However, we cannot make a definitive conclusion for several reasons. The first is that the vegetation index does not differentiate vegetation attributes, and therefore does not entirely represent kangaroo food resources, such as short grass and forbs. In fact, Letnic et al. (2009) showed that dingo exclusion can instead result in more grass cover, which would contradict our argument. Secondly, shrub encroachment is also linked to extirpation of the dingo (Gordon et al. 2017) south of the dingo fence, with shrub cover being 26–48% greater in areas where dingoes were rare. Woody weeds are more common in the dingoes-rare property (Cairns S.C., personal observation), which possibly increased vegetation indices. Yet, shrubs and woody plants also tend to be more perennial, which would not necessarily account for the greater ranges of productivity observed in the dingoes-rare property that could instead be caused by increased biomass of grasses and forbs. Further vegetation assessments are needed on-site from these properties to argue more definitively for or against the presence of higher productivity of kangaroo forage species in the dingoes-rare property. If vegetation differences are largely due to shrub encroachment, our results remain relevant as indicators of faster growth rates at lower densities (Plaisir et al. 2022).

Our “snapshot” of red kangaroo populations across the fence suggests that dingoes might have a substantial impact on the demographic composition and possibly also the growth patterns of red kangaroos, warranting further research including more sampling over time and across space. For example, we could not account for the impact of the longer-term sampling occurring on the two properties. While anecdotal reports suggest that commercial shooting began sometime in the 1950s inside the fence, previous research sampling over the years from both properties could have potentially created a ‘legacy effect’ in terms of artificially reduced densities. Differences in forage quality did not emerge from examining cranial shape, indicating that environmental differences are not of sufficient magnitude across the dingo barrier fence at this location to elicit clear shifts in morphology relating to feeding biomechanics, temperature control, water retention, or respiration (Johnston and Sharman 1976; Milne and O’Higgins 2002; Hadley et al. 2009; Nelson et al. 2017; Weisbecker et al. 2019; Mitchell et al. 2020). A lack of differences in cranial shape also supports the robustness of our age estimates. Molar progression is driven by propalinal occlusal pressure and is therefore subject to the amount a kangaroo chews (Sanson 1989; Barber et al. 2008). While differences in food toughness might accelerate molar progression due to the greater chewing loads required for tougher food (McArthur and Sanson 1988), the proximity of the two properties means that stark differences in food toughness are unlikely, given that such differences are usually more regional in scale (Mitchell et al. 2020). We also suggest a low likelihood that the kangaroos sampled for this study traveled from distant regions with different vegetation. While individual red kangaroos have been recorded traveling distances of over 100 km (Priddel et al. 1988b; Edwards et al. 1994), entire populations are not as nomadic, only traveling 20–30 km from their home ranges under more extreme conditions (Priddel et al. 1988a; Croft 1991), a distance falling within the dimensions of the two properties sampled (Fig. 1).

Fewer females and juveniles in the dingoes-common population support expectations from known patterns of dingo predation. Shepherd (1981) found that red kangaroo carcasses left by dingoes were usually juveniles, and that if carcasses were from adults, they were biased toward smaller females. The relatively small proportion of dingoes-rare males is also likely affected by selective shooting of larger males prior to our sample (Newsome 1977; Croft 1999). Generally, pouch young must be euthanized alongside shot mothers (Commonwealth of Australia 2008; AgriFutures Australia 2020), and shooters are encouraged to avoid shooting females with obvious signs of pouch young (Commonwealth of Australia 2008:8). Both populations had few males older than ~9 years, except for one individual from the dingoes-rare side of the dingo barrier fence. Although there is often a male bias in road kills among kangaroos and wallabies, there is no sex bias for *O. rufus* in this regard (Coulson 1997). A bias for large males in human hunting, culling, and harvesting is therefore a more likely explanation, and human aversions to killing juveniles and females might contribute to their greater representation in the dingoes-rare population. In South Australia, red kangaroos are only harvested commercially inside the fence, with shooters focusing on individuals heavier than about 40 kg (Cairns S.C., personal observation). This is because commercial harvesting in South Australia is mediated by population densities (South Australian Commercial Kangaroo Management Plan 2017) and the lower densities outside the fence have likely limited harvesting from there. If larger males have faster ontogenetic growth rates for male competition, harvesting might represent a selection pressure against faster growth rates. This would oppose any potential selection pressure for faster-growing males outside the fence contributed by dingoes.

Our data reveal a novel intraspecific relationship in the different allometric trajectories for relative pes length between males and females. This is not consistent with the interspecific trend for larger macropodid species to have relatively short pes lengths with increasing body mass (Richards et al., 2015). Despite many studies exploring ontogeny and growth patterns in various macropodiforms (Heinsohn 1968; Poole et al. 1982; Jarman 1989; Sinclair 1998; Johnson and Delean 2002), this feature has received little acknowledgment (but see Rose 1989). Our sexually dimorphic growth trajectories in pes lengths are so clear among mature individuals that the parameterized functions could potentially help predict sex from dry or paleontological specimens. While a relatively longer pes might be associated with male aggression in the form of powerful kicks (Ganslosser 1989), the similarly sexually dimorphic distributions of body weight and pes lengths also suggest potential mechanical importance in weight bearing during locomotion (Janis et al. 2014). Lengths of the leg and pes have been used previously as predictors of kangaroo age (Poole et al. 1982), and we suggest that these sexually dimorphic rates of pes development should be considered in future analyses.

Consistent with a previous study (Milne and O’Higgins 2002), we did not find clear evidence of sexual dimorphism in cranial shape. But like us, Milne and O’Higgins (2002) also noted a differential cranial growth where males of the same molar index had a larger cranium than females. Comparative biomechanical modeling of macropod crania has indicated greater robusticity in the skull of male *O. rufus* than other species (Mitchell et al. 2018). Therefore, outside of allometric-related effects, differences in cranial anatomy between the sexes might be less shape-related and more associated with greater bone volume, which would help to absorb blows to the head taken during agonistic behaviors between males.

In our single-year population “snapshot,” we found diverging population structures and slower ontogenetic growth rates among red kangaroos associated with generations of predator exclusion by the dingo barrier fence. These differences might arise via dingo predation, given we found greater vegetation biomass in the dingoes-rare zone; however, confirming that the differences in vegetation metrics indicate relative abundance of kangaroo forage species is required to draw stronger conclusions. Whether shifts in growth rates are a somatic or genetic response requires clarification, because genetic adaptation would have likely a greater impact on populations upon reexposure to dingo predation. The red kangaroo is particularly hardy, and the species will not likely be threatened removal of the dingo barrier fence. However, the developmental and demographic shifts identified here might also be present in other, more vulnerable native prey species, which could require attenuation periods to adjust to the return of a large predator. Such considerations will be necessary for developing sustainable conservation and management protocols, should the fence be moved or decommissioned.
