# Correction to: Differential developmental rates and demographics in Red Kangaroo (*Osphranter rufus*) populations separated by the dingo barrier fence

**DOI:** 10.1093/jmammal/gyad073

**Published:** 2023-07-08

This is a correction to: This is a correction to: D. Rex Mitchell, Stuart C. Cairns, Gerhard Körtner, Corey J. A. Bradshaw, Frédérik Saltré, and Vera Weisbecker, Differential developmental rates and demographics in red kangaroo (*Osphranter rufus*) populations separated by the dingo barrier fence, *Journal of Mammalogy*, 2023; gyad053, https://doi.org/10.1093/jmammal/gyad053

In the originally published version of this manuscript the Genus and species name of the Red Kangaroo (*Osphranter rufus*) was not italicized in the title.

This error has been corrected.

